# Zika knowledge and prevention practices among U.S. travelers: a large cross-sectional survey study

**DOI:** 10.1186/s12889-019-7533-3

**Published:** 2019-09-03

**Authors:** Maya Luetke, Oghenekaro Omodior, Erik J. Nelson

**Affiliations:** 1 Department of Epidemiology and Biostatistics, Indiana University School of Public Health, 1025 E. 7th Street, Suite 111, Bloomington, IN 47405 USA; 2 Department of Recreation, Park, and Tourism Studies, Indiana University School of Public Health, Bloomington, IN USA

**Keywords:** Zika virus, Zika knowledge, Zika prevention, Personal protective behaviors, Sexual transmission

## Abstract

**Background:**

The aim of this study was to investigate what factors predict knowledge about Zika transmission, symptomology, and treatment among U.S. travelers and, additionally, to evaluate how Zika knowledge influences the adoption of personal protective behaviors.

**Methods:**

Data were collected as part of a cross-sectional survey study using a probability-based internet panel of U.S. travelers in June 2017. We ran logistic regression models of factors predicting Zika knowledge (high vs. low) and of knowledge predicting adoption of personal protective measures.

**Results:**

We found that traveling to a Zika endemic country and travelers’ gender were both significantly predictive of higher Zika knowledge (odds ratio (OR): 1.48, 95% confidence interval (CI): 1.14–1.93 and OR: 1.44, 95% CI: 1.08–1.92), adjusting for age, race, education, income, and trip purpose. Additionally, among travelers to Zika endemic countries, individuals with higher Zika knowledge had significantly higher odds of engaging in preventive behaviors compared to those with lower knowledge. However, few travelers knew about the sexual transmission of Zika and adopted sexual prevention measures.

**Conclusions:**

Our findings suggest that there are gaps in knowledge about the risks and transmission of Zika and travelers with low knowledge are less likely to engage in the appropriate prevention methods. Significantly, few U.S. travelers have knowledge of the sexual transmission of Zika and, accordingly, there is less overall engagement with prevention measures for this transmission mechanism than for vector-borne transmission.

**Electronic supplementary material:**

The online version of this article (10.1186/s12889-019-7533-3) contains supplementary material, which is available to authorized users.

## Background

In the past few years, Zika virus has become a significant global public health concern with widespread transmission and potentially devastating consequences for children born to Zika-infected mothers. In 2015, a large-scale outbreak of Zika virus started in Brazil and spread rapidly throughout South America, Central America, and the Caribbean [1, 2]. Additionally, imported, sexually-transmitted, and a few isolated autochthonous cases were seen in the U.S., Europe, and elsewhere [3–6]. At the height of the outbreak in 2016, Zika virus and the associated “cluster[ing] of microcephaly and other neurological disorders reported in Brazil” prompted the World Health Organization (WHO) to declare an international public health emergency [7]. The severe clinical consequences in addition to the global connectedness of countries that allows for potential worldwide spread of Zika virus presents important public health challenges [8].

Zika virus is a vector-borne disease, predominantly transmitted by *Aedes* (*Stegomyia*) genus of mosquitoes, which includes the common and widely distributed *Aedes aegypti* [2]. However, sexual transmission of the virus has also been documented [9–11]. Furthermore, infection during pregnancy can have deleterious effects on the developing fetus, resulting in serious congenital malformations such as microcephaly, neurological sequelae, and Guillain–Barré syndrome [12]. Additionally, Zika is endemic in many parts of the world and the prevalence of antibody carriers is approximately 73% globally [13].

In the absence of vaccines or drug treatment for Zika virus infection, the interim guidance for Zika virus prevention provided by the U.S. Centers for Disease Control and Prevention (CDC), calls for public knowledge in relation to: (a) prevention of mosquito bites; (b) what to do before; during, and after to areas endemic for Zika virus; and (c) condom use for prevention of Zika transmission during sex [14]. Existing studies on Zika knowledge have focused on pregnant women and women of reproductive age [15–18], undergraduate students [19], and healthcare workers [20]. While these studies advance Zika virus research, very few studies have examined knowledge of Zika epidemiology, transmission, and prevention among travelers to Zika endemic regions [15, 21, 22]. It is critical to address this paucity of research, in part because, Zika virus infection is endemic in many parts of the world, and travelers constitute an important population in the spread of Zika virus into new regions and countries. The aim of this study is, therefore, to assess Zika knowledge among international travelers, predictors of Zika knowledge, and the association between Zika knowledge and use of personal protective measures. We hypothesize that (a) Zika knowledge will be high among U.S. travelers, (b) higher Zika knowledge will be associated with certain characteristics of U.S. travelers including traveling to a Zika endemic country, and (c) travelers with higher Zika knowledge will demonstrate greater engagement with and use of personal protective behaviors. This study adds to another study by our research team that was recently published in the same sample of U.S. travelers. The previous paper described the attributes and behaviors of the population and focused on the sexual transmission route of Zika virus [23]. Here, we go a step farther to model relationships between different predictors and level of Zika knowledge as well as the relationship between Zika knowledge and the adoption of recommended prevention behaviors.

## Methods

### Data collection

#### Study participants

In June of 2017, study participants were recruited from a probability-based internet panel of 6 million U.S. residents which is hosted by Qualtrics (Qualtrics, Provo, Utah, USA). [24, 25] Qualtrics is a global company that specializes in survey sampling and software development for survey research. Qualtrics has enlisted a large, worldwide panel of potential survey participants, of which, approximately 6 million are Americans. [24] A random sample of panel members who met our eligibility criteria were contacted by Qualtrics and invited to participate in the study. The eligibility criteria for study participation were: (a) adult men and women age ≥ 18 years of age; (b) residents of the U.S.; (c) spoke English; and (d) had a history of traveling outside of the U.S. We utilized a web-based survey panel design for our study because of the ability to collect large and diverse samples that are both cost-effective and time-efficient [26, 27]. Participation in the study was voluntary and all participants provided informed consent before beginning the survey. Qualtrics provided a small incentive to participants for their participation in the survey. As such, recruitment costs (including incentives) were $6.71 (USD) per completed survey. This study was approved by the Human Subjects Office at Indiana University (Protocol # 1705563810).

#### Survey questionnaire

In this cross-sectional survey study, study participants were asked to self-report demographics such as their age, gender, race/ethnicity, educational attainment, income, and health insurance status based on questions from the U.S. Census Bureau’s American Community Survey [28]. Respondents were also asked to report the countries outside of the U.S. to which they had traveled in the past year. These countries were categorized as Zika-endemic or non-Zika endemic based upon the WHO’s Zika virus classification table released in 2017 [29]. Finally, participants were asked to respond to questions to identify the signs and symptoms of Zika infection, risk factors for infection, modes of transmission, and preventative methods and practices to avoid infection. These Zika-related questions were taken directly from the WHO Knowledge, Attitudes and Practice survey about Zika virus [30]. We also assessed the ability of participants to correctly identify whether various preventive actions were effective or not. This information was derived from a multiple-answer question that asked, “How can you prevent Zika?”, and provided various effective and non-effective prevention methods as possible answers. Further, we assessed actual use of preventive actions among those traveling to Zika endemic countries. These included prevention methods such as use of mosquito nets, mosquito repellants, mosquito coils or fire, covering clothes, condoms, abstinence from sexual intercourse, removing standing water, spraying or fumigation, window or door screens, or avoiding Zika areas. We collected these data from a question which asked, “What action(s) have you take to prevent yourself / your household from getting Zika?”

### Statistical analysis

We performed the statistical analysis in a series of steps. First, we created an index of Zika knowledge that was a composite of several questions that aimed to assess knowledge and understanding of transmission, signs and symptoms, and risk factors among participants (see Additional file 1). Correct responses were scored as a one, and incorrect responses were scored as zero. The number of correct responses were summed for each participant (ranged from 1 to 27) and treated as an index of Zika knowledge. Then, the median of the index variable was used as the cut point to delineate two groups: high Zika knowledge (index scores of 14 to 27) and low Zika knowledge (index scores of 1 to 13). Hence, the high Zika knowledge category represents more comprehensive knowledge regarding Zika transmission, symptoms, and risks while the low Zika knowledge category represents a lesser understanding of Zika transmission, symptoms, and risks.

Next, we ran logistic regression models in order to determine which variables had significant predictive power on having higher Zika knowledge. In order to show the relative impact of each factor, we present 3 logistic regression models where variables were added in a stepwise manner until we reached the full model. Specifically, our first model examined the relationship between the outcome variable of Zika knowledge (high/low) and traveling to a Zika endemic country. The second model was the same as the first with the addition of gender as a predictor. The third and final model also accounted for the effects of the aforementioned demographic covariates in order to control for potential confounding. In all models, we exponentiated the model coefficients (i.e., exp.[B]) to present odds ratios (ORs) with their corresponding 95% confidence intervals (CIs).

In our final analysis step, we conducted a variety of analyses among a subset of the study population who had traveled to a Zika endemic country in the past 12 months. First, we examined whether overall Zika knowledge was predictive of engagement in personal protective measures (i.e., using bed nets, mosquito repellant or contraceptives) through a series of bivariate logistic regressions where each preventative action was associated with the Zika knowledge index. Again, we exponentiated the model coefficients to present ORs with their corresponding 95% CIs. Second, we described the number (and percentages) of individuals in this subsample that perceived different prevention methods as being efficacious. All statistical analyses were conducted using R version 3.4.1 (“Single Candle”) and SAS 9.4 (Cary, NC) [31, 32].

## Results

**Fig. 1:**
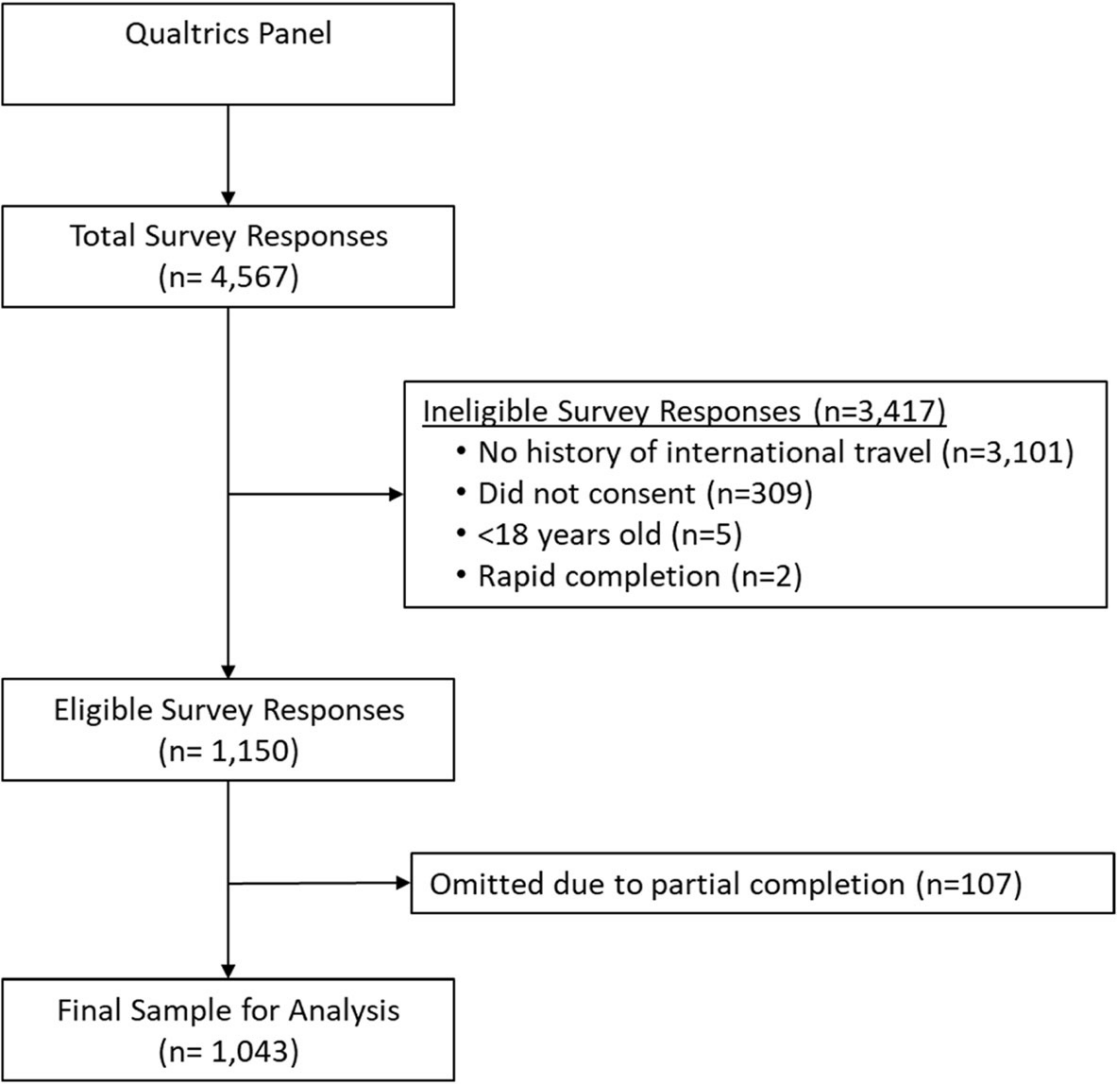
Study recruitment diagram

A total of 4567 individuals were contacted for potential participation in this study. Of these, 309 (9%) did not consent to participate, 5 (0.2%) were ineligible because they we less than 18 years of age, 3101 (90.2%) were ineligible because they reported no history of international travel, and 2 (0.06%) completed the entire survey in less than 30 s and were omitted. Of the remaining 1150 responses, 107 (9.3%) were omitted due to partial survey completion (less than 50% of the survey was completed). Accordingly, the final analytic sample size was 1043. This was comprised of 22.8% of all those that were invited to participate and 90.6% of those who met the eligibility criteria for the study. Figure 1 shows a summary of overall participant recruitment for the study.

Demographically, the study population had a mean age of 36.1 years (SD = ±13.8). Most of the sample (55%) had some college education (i.e. some college work toward a bachelor’s degree but not yet graduated or had obtained an associate degree) or were college graduates (i.e. had obtained a bachelor’s degree). Nearly three quarters were employed and the majority had an annual household income of over $50,000 (please see Table 1 from Nelson et al. 2019). [23]

**Table 1:** Predictors of high Zika knowledge among U.S. travelers (*N* = 1043)

Characteristic	Model 1		Model 2		Model 3	
	OR	95% CI	OR	95% CI	OR	95% CI
Visited Zika endemic country in last year						
No	1		1		1	
Yes	1.42*	(1.11–1.82)	1.38*	(1.07–1.76)	1.48*	(1.14–1.93)
Gender						
Male			1		1	
Female			1.47*	(1.13–1.93)	1.44*	(1.08–1.92)
Purpose of travel						
Business/ Leisure					1	
Vacation					0.82	(0.57–1.18)
Age, years						
18–29					1	
30–39					0.85	(0.61–1.17)
40–49					0.69	(0.45–1.04)
50–59					0.59*	(0.36–0.96)
60–69					0.81	(0.45–1.46)
70+					0.84	(0.37–1.91)
Race						
Caucasian					1	
African American					0.58*	(0.38–0.88)
Other					1.16	(0.80–1.68)
Education						
Less than high school graduate					1	
High school graduate					1.19	(0.34–4.17)
Some college or associate degree					1.71	(0.50–5.86)
College graduate/bachelor’s degree					1.66	(0.48–5.66)
Annual household income, US $						
Less than 15,000					1	
15,000–24,999					1.21	(0.54–2.70)
25,000–34,999					1.40	(0.67–2.91)
35,000–49,999					1.36	(0.68–2.75)
50,000–74,999					2.18*	(1.12–4.26)
75,000–99,999					1.42	(0.71–2.84)
100,000 or more					2.27*	(1.15–4.47)

*Indicates significant finding at an a priori α value of 0.05

Our model showed that traveling to a Zika endemic country and gender were both predictive of Zika knowledge (Table 1). Those traveling to a Zika endemic country had 48% greater odds of having high Zika knowledge compared to those not traveling to a Zika endemic country (OR: 1.48, 95% CI: 1.14–1.93). Additionally, women had higher odds of having high Zika knowledge compared to men (OR: 1.44, 95% CI: 1.08–1.92). In our final model, we adjusted for age, race, education, income, and trip purpose (Table 1).

Among the 460 travelers that had been to a Zika endemic country in the past 12 months, individuals with higher Zika knowledge had significantly higher odds of engaging in preventive actions compared to those with lower Zika knowledge (Table 2). Compared to people with low Zika knowledge, those with high Zika knowledge had varying increased odds of engaging in prevention actions. Significantly, those with high Zika knowledge were about 5 to 6 times as likely to adopt sexual transmission preventive actions, such as condom use and abstinence (Table 2). Table 2 also shows that those with higher Zika knowledge have significantly higher odds of engaging in vector-focused prevention of Zika. Travelers with high Zika knowledge had significantly increased odds of adopting the following effective prevention behaviors compared to travelers with low Zika knowledge: use of mosquito repellant (OR: 2.69, 95% CI: 1.82–4.01), mosquito coils or fire (OR: 1.75, 95% CI: 1.10–2.81), wearing covering clothes (OR: 2.53, 95% CI: 1.64–3.94), removing standing water (OR: 2.79, 95% CI: 1.75–4.52), spraying or fumigating for mosquitoes (OR: 2.18, 95% CI: 1.21–4.05), and putting screens on windows and doors (OR: 3.87, 95% CI: 2.26–6.89).

**Table 2:** The effect of high Zika knowledge compared to low on preventive actions among Americans traveling to Zika endemic countries (*N* = 460)

Adopted preventive action	Participant Zika Knowledge		Comparison of Zika Knowledge (high vs. low)	
	High Zika Knowledge N (%)	Low Zika Knowledge N (%)	OR^a^	95% CI
I. Effective preventive actions				
Vector-related prevention				
Window and door screens	58 (25.6)	19 (8.2)	3.87*	(2.26–6.89)
Other: Avoid Zika areas	3 (1.3)	1 (0.4)	3.11	(0.39–63.07)
Remove standing water	68 (30.0)	31 (13.3)	2.79*	(1.75–4.52)
Mosquito repellant	107 (47.1)	58 (24.9)	2.69*	(1.82–4.01)
Covering clothes	78 (34.4)	40 (17.2)	2.53*	(1.64–3.94)
Spray/fumigate	35 (15.4)	18 (7.7)	2.18*	(1.21–4.05)
Mosquito coils/fire	55 (24.2)	35 (15.5)	1.75*	(1.10–2.81)
Mosquito nets	39 (17.2)	34 (14.6)	1.21	(0.74–2.01)
Sexual-related prevention				
Abstinence	17 (7.5)	3 (1.3)	6.21*	(2.05–26.85)
Condoms	30 (13.2)	6 (2.6)	5.76*	(1.43–6.90)
II. Non-effective preventive actions				
Non-barrier contraception	9 (4.0)	4 (1.7)	2.36	(0.76–8.83)

^a^OR indicates odds ratio from a bivariate logistic regression. The OR can be interpreted as the odds of engaging in the preventive action for high Zika knowledge participants compared to the odds of engaging in the preventive action for low Zika knowledge participants

*Indicates significant finding at an a priori α value of 0.05

Additionally, among travelers to Zika endemic countries, the most commonly recognized prevention methods against Zika virus were; (a) mosquito repellant (87.2%); (b) wearing covering clothes (75.2%); and (c) mosquito nets (64.6%) (Table 3). Overall, approximately 37 to 87% of participants were able to correctly identify effective vector-focused prevention methods. In contrast, only 27 to 35% of participants were able to correctly identify effective methods for the prevention of the sexual transmission of Zika virus (Table 3).

**Table 3:** Knowledge of the efficacy of various preventive actions among Americans traveling to Zika endemic countries (*N* = 460)

Action	N (%)
I. Effective prevention actions	
Vector-related prevention	
Mosquito repellant	401 (87.2)
Covering clothes	346 (75.2)
Mosquito nets	297 (64.6)
Window and door screens	274 (59.6)
Mosquito coils/fire	270 (58.7)
Remove standing water	255 (55.4)
Spray/fumigate	171 (37.2)
Sexual-related prevention	
Condoms	162 (35.2)
Abstinence	126 (27.4)
II. Non-effective prevention actions	
Clean household	141 (30.7)
Drink clean water	127 (27.6)
Non-barrier contraception	47 (10.2)

## Discussion

International travel has increased steadily and dramatically over the past several decades with a record 1.2 billion international arrivals in 2016 [33]. As a consequence of this human movement and migration, infectious diseases have been able to take root in new geographic regions and there have been multiple pandemics in the past several years [34, 35]. Infectious diseases also cause significant morbidity in travelers [36]. Accordingly, disease knowledge, appropriate preparation, and prevention actions for travel is important for the prevention of infection and transmission of infectious agents. Our findings show that among travelers there are gaps in knowledge about the risks and transmission of Zika virus and that travelers with low knowledge are much less likely to engage in the appropriate prevention methods when traveling to a Zika endemic country. This is especially significant because Zika has a vast potential for spread with many regions supporting the arthropod vector that have not yet seen or have seen isolated imported or autochthonous transmission [3, 14]. The fact that imported or autochthonous Zika cases have been reported in the U.S. (in California, Texas, and Florida), Europe, and elsewhere indicate the potential spread of the virus associated with travel [4–6]. Therefore, travelers, especially those traveling to Zika endemic countries, are an important population to target with future Zika knowledge campaigns.

Additionally, there seems to be an overall lack of knowledge of the sexual transmission of Zika. As such, fewer travelers to a Zika endemic country perceived the personal protective measures of condom use and abstinence to be effective compared to other personal protective measures. Although general knowledge of the sexual transmission of Zika was rather low, our findings suggest that those with higher Zika knowledge do have knowledge of the sexual transmission of Zika virus. Accordingly, the disparity in the adoption of sexual transmission preventive actions is particularly stark between travelers to Zika endemic regions with low knowledge and high knowledge. This is significant for several reasons. First, this gap in knowledge should be addressed in public information dissemination efforts by emphasizing the sexual transmission risks of Zika. Second, partners of Zika infected individuals may not realize they are at risk. In fact, sexual transmission risks of Zika may be hugely underestimated. Though some studies have suggested that sexual transmission of Zika is extremely low (nearing 1%) [37, 38], it is difficult to separate this transmission route from vector-borne transmission in Zika endemic regions and thus, these estimates may not be reflective of actual numbers of sexually contracted cases. A recent study in monkeys indicates that sexual transmission may be much higher than experts previously thought—with 11 out of 16 (or 69%) sexually exposed monkeys becoming infected with Zika [39]. With the potential high infection rate from sexual exposure, it is ever more important to disseminate information about the sexual transmission risks and the importance of sexual prevention methods.

Finally, mosquito nets were employed with greater likelihood by those with higher Zika knowledge but to a lesser degree than the other vector-focused prevention measures. Insecticide treated mosquito nets have been trumpeted as a highly effective in prevention of arthropod-borne diseases, particularly malaria, and there has been a large global movement to both encourage the their use and to distribute them widely with the goal of universal coverage in high risk regions [40]. The fact that people are already utilizing mosquito nets to prevent other arthropod-transmitted infections and the fact that the primary Zika vector, the *Aedes* mosquito, typically bites during the morning and evenings rather than during the night may account for this smaller difference between those with high knowledge and those with low knowledge [41]. In addition to accessibility, this preventive measure also represents one of the most simple and inexpensive methods of prevention**.**

There are some limitations to this study. First, there is a lack of causal inference due to the cross-sectional design of the study. Second, participants were recruited from a survey panel hosted by Qualtrics and may not be representative of the general U.S. population. Third, survey responses were self-reported by people through the internet and may be subject to under or over reporting of individuals’ knowledge of Zika transmission, signs and symptoms, and risk factors. However, other internet-based studies have shown increased self-disclosure and reporting with online surveys, which may reduce potential response biases (e.g., interviewer bias or social desirability) [42, 43]. Finally, the inclusion criteria of being able to communicate in the English language, though a necessary requirement for our study, may introduce some selection bias as it may have excluded American travelers who frequent Zika endemic regions and do not speak English. However, we expect that these numbers are small since those residing in the U.S. generally have at least elementary English language abilities. Despite these limitations, our study finding that few U.S. travelers have knowledge of the sexual transmission of Zika has important public health implications. Namely, this finding can inform the planning of tailored health messaging for Zika prevention among U.S travelers to both Zika endemic and non-endemic international locations.

## Conclusions

Our results reveal that traveling to a Zika endemic country and being of the female gender increased the odds of having higher Zika knowledge. However, our findings also suggest that there are gaps in knowledge about the risks and transmission of Zika and travelers with low knowledge are less likely to engage in the appropriate prevention methods. Specifically, there is little knowledge of the sexual route of Zika virus transmission among U.S. travelers. This is particularly important, as new research among monkeys indicates that sexual transmission may be more common than previously thought. Without sufficient awareness of the sexual transmission of Zika, travelers will be ill-equipped to adopt the appropriate personal protective measures to prevent the sexual spread of this virus.

## Additional files

Additional file 1: Survey questions used for the construction of the Zika knowledge index. (DOCX 17 kb)

## Data Availability

The data that underpin the findings of the current study are available from the corresponding author [ML] on reasonable request.

## Abbreviations

CDC: Centers for Disease Control and Prevention
CI: Confidence Interval
OR: Odds Ratio
U.S.: United States
WHO: World Health Organization
