# Supplementary material for: Zika knowledge and prevention practices among U.S. travelers: a large cross-sectional survey study
Source: BMC Public Health. 2019 Sep 3;19:1217. doi: 10.1186/s12889-019-7533-3 (PMC6724273; doi:10.1186/s12889-019-7533-3)
Supplement: Supplementary file 1 — Survey questions used for the construction of the Zika knowledge index. (DOCX 17 kb) [file 12889_2019_7533_MOESM1_ESM.docx]

**ADDITIONAL FILE 1:**

**Survey questions used for the construction of the Zika knowledge index**

| **Question** | **Correct answers** | **Index Points** | **Variable used for analysis** |
| --- | --- | --- | --- |
| **1. Who can get Zika? (Select all that apply)** |  |  |  |
| Adult men |  | 0 |  |
| Adult women |  | 0 |  |
| Women of child bearing age (15-49) |  | 0 |  |
| Boys |  | 0 |  |
| Girls |  | 0 |  |
| Pregnant women |  | 0 |  |
| Health workers |  | 0 |  |
| Everybody can get Zika | Correct | 1 | zikaWC$z_everybody |
| **2. What causes Zika? (Select all that apply)** |  |  |  |
| Mosquitos | Correct | 1 | zikaWC$c_mosq |
| Polluted water |  | 0 |  |
| Sexual intercourse | Correct | 1 | zikaWC$c_sex |
| Spraying / fumigating |  | 0 |  |
| Pesticides / insecticides |  | 0 |  |
| Virus | Correct | 1 | zikaWC$c_virus |
| Breast milk | Correct | 1 | zikaWC$c_brmilk |
| Vaccinations |  | 0 |  |
| **3. How does a person get Zika? (Select all that apply)** |  |  |  |
| Mosquito bite | Correct | 1 | zikaWC$g_mosq |
| Drinking polluted water |  | 0 |  |
| Through sexual intercourse | Correct | 1 | zikaWC$g_sex |
| Through coughing and sneezing (e.g., airborne) |  | 0 |  |
| From a virus | Correct | 1 | zikaWC$g_virus |
| From breast milk | Correct | 1 | zikaWC$g_brmilk |
| From vaccinations |  | 0 |  |
| From spraying/fumigation |  | 0 |  |
| From pesticides/insecticides |  | 0 |  |
| From a blood transfusion | Correct | 1 | zikaWC$g_blood |
| From mother to child transmission | Correct | 1 | zikaWC$g_mtochild |
| Other (ALL ARE "I DON'T KNOW") |  | 0 |  |
| **4. What are the signs and symptoms of Zika? (Select all that apply)** |  |  |  |
| Fever | Correct | 1 | zikaWC$s_fever |
| Headache | Correct | 1 | zikaWC$s_headache |
| Rash | Correct | 1 | zikaWC$s_rash |
| Joint pain | Correct | 1 | zikaWC$s_joints |
| Sickness | Correct | 1 | zikaWC$s_sickness |
| Conjunctivitis (red eyes) | Correct | 1 | zikaWC$s_conj |
| Diarrhea |  | 0 |  |
| Hemorrhage / bleeding |  | 0 |  |
| Other (ALL ARE "I DON'T KNOW") |  | 0 |  |
| **5. Does everybody who gets Zika show symptoms?** |  |  | zikaWC$symptoms01 |
| Yes, Maybe, Don't know |  | 0 |  |
| No | Correct | 1 |  |
| **6. Can you prevent Zika?** |  |  | zikaWC$prevent01 |
| Yes, Maybe | Correct | 1 |  |
| No, Don't know |  | 0 |  |
| **7. Is there treatment for Zika?** |  |  | zikaWC$treatment01 |
| Yes, Maybe, Don't know |  | 0 |  |
| No | Correct | 1 |  |
| **8. What individuals or groups are more at risk of harm from Zika? (Select all that apply)** |  |  |  |
| Pregnant women | Correct | 1 | zikaWC$r_preg |
| Women of childbearing age (15-49 years) |  | 0 |  |
| Adolescents and unmarried women (15-24 years) |  | 0 |  |
| People with disabilities |  | 0 |  |
| Children |  | 0 |  |
| Elderly people |  | 0 |  |
| Nobody is more at risk |  | 0 |  |
| **9. If a pregnant woman has Zika, what are the risks she faces? (Select all that apply)** |  |  |  |
| She may be sick | Correct | 1 | zikaWC$pr_sick |
| She is at risk of miscarriage | Correct | 1 | zikaWC$pr_misc |
| She may have difficulty giving birth |  | 0 |  |
| She is at risk from illegal and/or unsafe termination of pregnancy |  | 0 |  |
| **10. If a pregnant woman has Zika, what are the risks for the fetus / baby? (Select all that apply)** |  |  |  |
| Risk of not growing or developing normally in the womb | Correct | 1 | zikaWC$b_grow |
| Risk of miscarriage | Correct | 1 | zikaWC$b_misc |
| Risk of being born prematurely |  | 0 |  |
| Risk of being stillborn |  | 0 |  |
| Risk of being born with Microcephaly | Correct | 1 | zikaWC$b_micro |
| Risk of being born with a disability |  | 0 |  |
| **11. Do you think there is a link between Zika and Microcephaly?** |  |  | zikaWC$micro01 |
| Yes, Maybe | Correct | 1 |  |
| No, Don't know |  | 0 |  |
| **TOTAL POSSIBLE SCORE** |  | **27** |  |
